# Metabolomic Assay, Computational Screening, and Pharmacological Evaluation of *Caulerpa racemosa* as an Anti-obesity With Anti-aging by Altering Lipid Profile and Peroxisome Proliferator-Activated Receptor-γ Coactivator 1-α Levels

**DOI:** 10.3389/fnut.2022.939073

**Published:** 2022-07-14

**Authors:** Happy Kurnia Permatasari, Fahrul Nurkolis, Hardinsyah Hardinsyah, Nurpudji Astuti Taslim, Nindy Sabrina, Faisal Maulana Ibrahim, Jodi Visnu, Dian Aruni Kumalawati, Sri Awalia Febriana, Toto Sudargo, Melvin Junior Tanner, Isma Kurniatanty, Vincentius Mario Yusuf, Ronald Rompies, Muhammad Rahimi Bahar, Holipah Holipah, Nelly Mayulu

**Affiliations:** ^1^Department of Biochemistry and Biomolecular, Faculty of Medicine, Brawijaya University, Malang, Indonesia; ^2^Department of Biological Sciences, Sunan Kalijaga State Islamic University, Yogyakarta, Indonesia; ^3^Department of Applied Nutrition, IPB University, Bogor, Indonesia; ^4^Department of Clinical Nutrition, Faculty of Medicine, Hasanuddin University, Makassar, Indonesia; ^5^Department of Nutrition, Dietetics and Food, Faculty of Medicine, Nursing and Health Sciences, Monash University, Melbourne, VIC, Australia; ^6^Pharmaceutical Analysis and Medicinal Chemistry, Universitas Padjadjaran, Sumedang, Indonesia; ^7^Faculty of Medicine, Public Health and Nursing, Universitas Gadjah Mada, Yogyakarta, Indonesia; ^8^Department of Nutrition, Faculty of Public Health, University of Indonesia, Depok, Indonesia; ^9^Faculty of Medicine, Universitas Brawijaya, Malang, Indonesia; ^10^Faculty of Medicine, Sam Ratulangi University, Manado, Indonesia

**Keywords:** PGC-1α, obesity, *Caulerpa racemosa*, nutraceuticals, lipid profile

## Abstract

Obesity is associated with an accelerated aging process, which prevents healthy aging. Both obesity and aging were manifested in the peroxisome proliferator-activated receptor-γ coactivator α (PGC-1α) level. These studies fulfill the scientific gap in assembled pharmacological activity assay of *Caulerpa racemosa* done in a previous preclinical trial. Six major compounds from sea grape (*C. racemosa*) extract were evaluated using an *in silico* approach against human pancreatic *lipase*, *a-glucosidase*, and *a-amylase* to predict prospective anti-obesity candidates. The *lipase* inhibitory activity of the extract reached 90.30 ± 0.40%, 1.75% lower than orlistat. The *a-amylase* inhibitory assay of the extract was 84.07 ± 5.28%, while the inhibitory activity against *a-glucosidase* was 81.67 ± 1.54%; both were lower than acarbose. We observe the effect of *C. racemosa* extract as anti-obesity with anti-aging by evaluating the obesity parameters in the human body for a 4-week period. There was a significant decrease in blood glucose, total cholesterol, low-density lipoprotein (LDL), triglycerides (TG), waist circumference, waist-hip ratio, and body weight (*p* < 0.05); PGC-1α and high-density lipoprotein (HDL) increased significantly (*p* = 0.000), in Group B when compared with Group A. Our study revealed that sea grape extract is a potent anti-obesity with an anti-aging reagent that does not produce any significant adverse effects.

## Introduction

The prevalence of obesity and central obesity in Indonesia’s adult population is 23.1 and 28%, respectively (1). Based on the Indonesian National Health Survey (2018), North Sulawesi province ranked the first highest province with the prevalence of obesity in adults aged >18 years (1). Moreover, the proportion of central obesity at the age of ≥15 years in North Sulawesi province reached 42.5% (1). Manado is one of the largest cities and the center of the capital of North Sulawesi (2); therefore, this study is an important effort to reduce obesity rates in one of the provinces with high obesity rates in Indonesia. Obesity is related to noncommunicable diseases that are closely related to chronic diseases and aging (3). Another study showed that obesity plays a large role in reducing the productive period by 6–10 years (4). In addition, in Indonesia, obesity also accounts for 8–16% of the national health cost budget, and in 2016, the total (direct and indirect) impact of obesity is estimated at 2–4 billion dollars (5). Moreover, people with obesity also often undergo psychological pressure, such as bullying (6, 7). The rise of lipid disorders in the world is associated with a variety of health problems, including diabetes, heart disease, chronic diseases, aging, and even cancer (8–10). Therefore, several solutions must be explored to accelerate the treatment of obesity, chronic diseases, and aging, such as the exploration of supplement candidates and functional foods that are natural from this rich Indonesian natural ingredients that can be preventive and therapeutic for the reduction of obesity, through various complex mechanisms including improvement of lipid profile and improvement of metabolic gene regulation.

One gene that regulates metabolism in the body is PGC-1α which even has the nickname “a key regulator of energy metabolism” through its complex role (11). *Peroxisome proliferator-activated receptor (PPAR)-γ coactivator 1 alpha (PGC-1α)* plays a role in many substantial metabolic processes and energy homeostasis and promotes muscle tissue remodeling, which is oxidative and less glycolytic (11). In conjunction with PGC-1α on mitochondrial respiration (regulating respiration) in muscle cells, this co-activator also induces gene expression for the insulin-sensitive glucose transporter (Glut-4) and increases glucose uptake (12, 13). PGC-1α also induces brown adipose tissue (BAT), which usually declines with aging (12). BAT, which is a brown adipocyte, contains many small droplets and a much higher number of mitochondria (containing iron), which gives the tissue its color (14). Brown fat also contains more capillaries than white fat, and this supplies tissues with oxygen and nutrients and distributes the heat generated throughout the body (14). Noticing the function of PGC-1α in the thermogenic brown adipose tissue (BAT) program, the regulation of Glut-4 and mitochondrial oxidation in muscle, and the dominant role of PGC-1α in hepatic gluconeogenesis along with the promotion of muscle tissue remodeling activity, all of which suggest that this co-activator can be a target for anti-obesity, antidiabetic, and anti-aging supplements (chronic diseases and aging) (11–13). In addition to the prevalence in adipose cells and skeletal muscle cells, the review study by Kadlec et al. demonstrated that PGC-1α is also expressed in blood flow independently of combating the ROS production by increasing the ATP/ADP translocase activity (15). In line with that, research by Zhu et al. used blood serum samples to determine PGC-1α levels in diabetes mellitus patients with myocardial infarction (16).

Sea grapes (*Caulerpa racemosa*) or lawi-lawi (local Indonesian term) is a species of green algae classified as the *Caulerpaceae* family that has been found in the waters around Sulawesi (17). Sea grapes are harvested intensively because they are an important source of macronutrients and micronutrients, especially in East and Southeast Asia (commercially grown in ponds and consumed in parts of the Philippines, Indonesia, and Vietnam) as the main source of traditional diets (18), although it is still used for health products. Several studies have shown that sea grapes contain several bioactive components, such as bioactive peptides, polysaccharides, polyphenols, flavonoids, and antioxidants (19–22). Additionally, sea grapes contain high antioxidant levels and may have potential as functional foods or nutraceuticals (20, 21, 23, 24). Doses of 150 mg/kg BW (30 mg/200 g BW) sea grape extract in our previous preclinical trial showed that it could improve blood glucose (BG), total cholesterol (TC), and serum PGC-1α levels in rats fed a diet high in fat and cholesterol (25). In addition, sea grapes also have hepatoprotective activity (aka non-toxic) in diabetic mice (22). Previous research (25), is an *in vivo* (preclinical) study that has not represented the benefits or efficacy of sea grape extract on variables tested in humans. Therefore, this clinical trial was carried out to support the pharmacological effect of sea grape extract-antioxidant on BG, TC, and PGC-1α levels in obese men for 4 weeks using a randomized-double blind controlled clinical trial (RCT). To support the RCT results, the study also explored the bioactive metabolites of *C. racemosa* through metabolomic profiling studies and looked at the activity of major compounds against *lipase*, *α-glucosidase*, and *α-amylase* enzymes computationally or *in silico* study and *in vitro*.

## Materials and Methods

### Extract of Sea Grapes Production

Fresh sea grapes (*C. racemosa*) are collected in the shallows (5–10 m above sea level) of the Mantehage seawater, north of Sulawesi, Indonesia. Botanical identification and authentication are confirmed in the Department of Pharmacology, Faculty of Mathematics and Natural Sciences, Sam Ratulangi University, Indonesia. Specimens are collected for future reference. Sea grapes (whole-body) are thoroughly rinsed with water, air-dried at room temperature, and baked at 40°C, then smoothed with an electric grinding. Furthermore, in the extract preparation, coarse powder (1,000 g) is macerated with 96% ethanol for 72 h with each extraction carried out in triple, resulting in a yield of 34%. The extract is roughly filtered with Whatman 41 filter paper. The total filtrate is glued and evaporated at 40°C in the RV 8 IKA rotary evaporator under reduced pressure (100 mbar) for 90 min and evaporated in an oven at 40°C to produce the powder extract. The extract is stored in a refrigerator at a temperature of 10°C until used in the research. The extract powder is encapsulated.

### Metabolomic Assay

The untargeted metabolomics profiling test of sea grape extract was carried out using the liquid chromatography–high-resolution mass spectrometry (LC-HRMS) method at the Laboratorium Sentral Ilmu Hayati (LSIH; ISO 9001:2008 and ISO 17025:2005; Central Laboratory of Life Sciences; Brawijaya University, Indonesia) testing services, with the test number 041/LSIH-UB/LK/II/2022. A total of 50 μl extract sample is diluted using 96% ethanol up to a final volume of 1,500 μl. The solution is vortexed at 2,000 rpm for 2 min and then span-down at 6,000 rpm for 2 min. The supernatant was taken and then filtered using a 0.22-μm syringe filter and injected into the vial. The sample in the vial is ready to be inserted into an autosampler and then injected into LC-HRMS. LC-HRMS uses a HPLC Thermo Scientific Dionex Ultimate 3000 RSLCnano system with a microflow meter. Solvents: A = 0.1% formic acid in water and B = 0.1% formic acid in acetonitrile. Analytical column: Hypersil GOLD aQ 50 × 1 mm × 1.9 μ particle size. Analytical flow rate: 40 μL/min. Flow gradient: run time: 30 min. Column oven: 30°C. High-resolution mass spectrometer using a Thermo Scientific Q Exactive, Full scan at 70,000 resolution, data-dependent MS2 at 17,500 resolution, run time: 30 min, polarity: positive and or negative. Processing data software: Compound Discoverer with mzCloud MS/MS Library. The results of the metabolites are shown in Supplementary 2. Major compounds that were successfully identified were continued *in silico* or molecular docking tests on *lipase* enzymes, *α-glucosidase*, and α*-amylase* computationally.

### Molecular Docking Simulation

#### Hardware and Software

Hardware (Hardware) used is Lenovo Thinkpad X240 with Intel^®^ Core™ i3-4010U CPU 1.70 GHz specifications, GPU Intel HD Graphics with 4.00 GB RAM. Equipped with software (Software) ChemOffice 2010 and ChemDraw Ultra 12.0, AutoDock tools (version 4.2), Ligandscout, BIOVIA Discovery, and Protein Data Bank, https://www.rcsb.org.

#### Preparation of Ligands and Targets

There were six compounds from the ethanolic extract of *C. Racemosa*, which were used as test ligands (BGPA, ITPA, DPA, betaine, choline, and dibutyl phthalate). The entire structure is drawn in 2D using ChemDraw Ultra 12.0, and then converted into 3D and optimized through the MM2 function. The target protein used are human pancreatic lipase (PDB ID: 1LPB), alpha-amylase (PDB ID: 2QV4), and alpha-glucosidase (PDB ID: 3L4Y) which were downloaded from the Protein Data Bank *via* the website https://www.rcsb.org/. Kollman charges were added to the receptors while Gasteiger charge was applied to the ligands.

#### Validation of Molecular Docking

The molecular docking validation method is carried out by re-docking the original ligand to the target pocket with certain grid coordinates using AutoDock tools (Version 4.2). The RMSD (root-mean-square deviation) of the ligand position after the re-docking procedure must be lower than 2.0 Å (Table 2).

**TABLE 1:** Six major compounds based on metabolomic assay of *C. racemosa* extract.

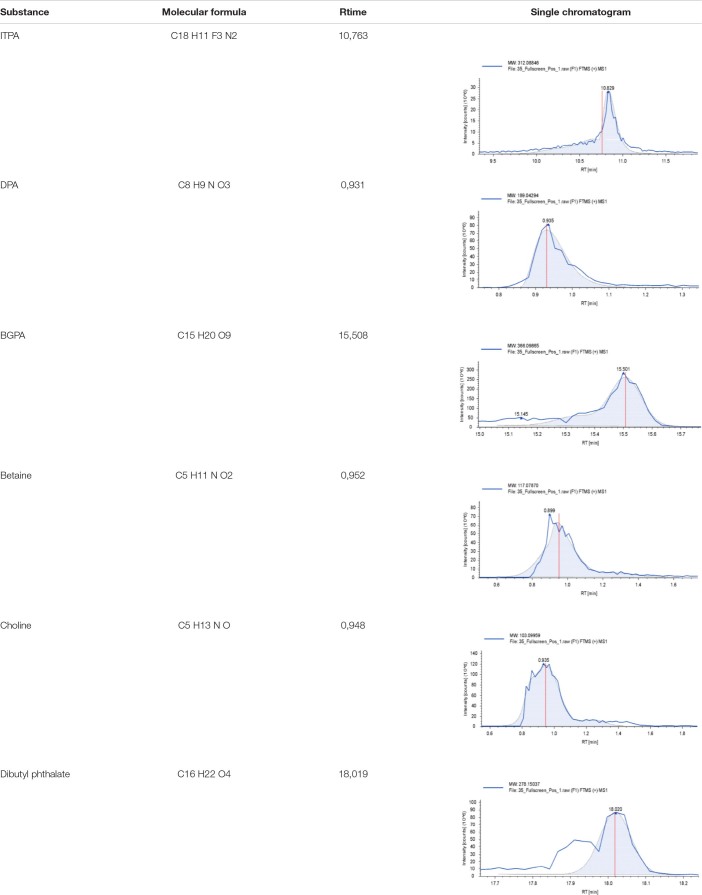

**TABLE 2:** Validation of molecular docking simulation.

No.	Drug target	PDB ID	Docking site (x;y;z)	Docking area (x.y.z)	RMSD (Å)	ΔG (kcal/mol)	Numb in cluster (/100)	Judgment (<2 Å)
1	Human pancreatic *lipase*	1LPB	4.448, 27.955, 49.675	40 × 40 × 40	1.86	−6.3	21	Valid
2	Human pancreatic *α-amylase*	2QV4	12.942, 47.170, 26.200	42 × 40 × 40	1.83	−9.96	20	Valid
3	Human pancreatic *α-glucosidase*	3L4Y	−1.542, −19.201, −21.043	42 × 40 × 40	1.63	−5.43	36	Valid

#### Molecular Docking Simulation

Grid and docking parameters were created by applying the results of docking validation as shown in Table 2. The result was written in a *dlg file for each final structure of docking conformation, and the ligand-receptor interaction was analyzed by Discovery Studio 2016.

### *In vitro* Studies

#### Antioxidant Activity (2,2-Diphenyl-1-Picrylhydrazyl Assay)

Antioxidant activity is determined using 2,2-diphenyl-1-picrylhydrazyl (DPPH; C_18_H_12_N_5_O_6_) (26). Each sample consisting of 1 ml was placed in microplate 96 and an aliquot (100 μL) of DPPH 0.3 mM was added, then incubated for 30 min in a darkroom. Sample absorption was measured using an ELISA reader at wavelength 517 nm. The antioxidant activity is calculated using Equation 1 as follows:


$$Inhibition\left(\%\right)=\frac{\left(A\,0-A\,1\right)}{A\,0}\times 100\%$$

where, A0, blank absorption; A1, standard or sample absorption.

The results of the antioxidant activity test against DPPH resulted in a value of 45.66 ± 0.55% (Triplicates/Triplo).

**FIGURE 1:**
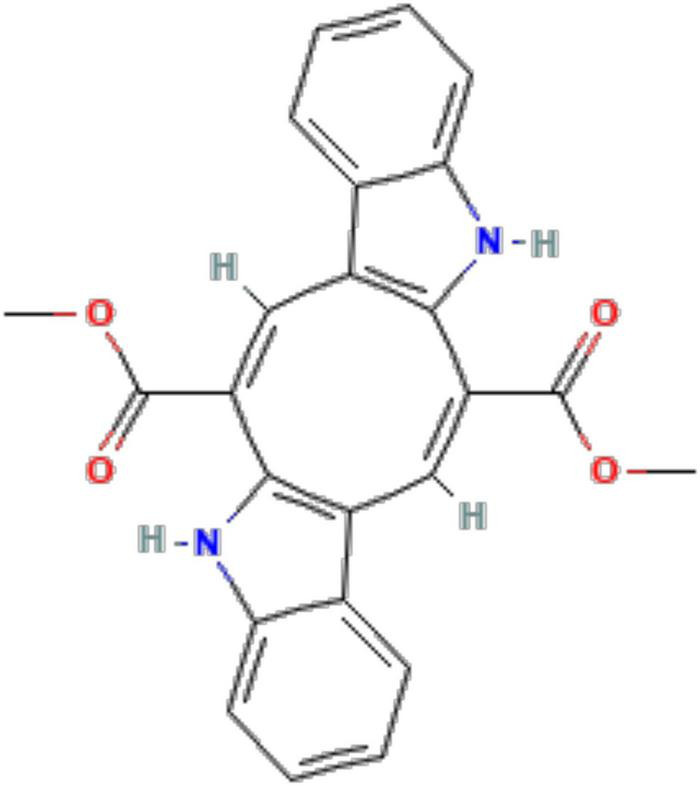
Chemical structure depiction of caulerpin (PubChem ID: 5326018).

Caulerpin (Figure 1; PubChem ID: 5326018) with the molecular formula: C_24_H_18_N_2_O_4_ is a typical secondary metabolite compound belonging to the algae genus *Caulerpa* sp. and is a bioactive compound from a group of alkaloid compounds that also function as antioxidants (Figure 1) (27). Therefore, a further test on caulerpin antioxidant levels in the extract (sample) was carried out using liquid chromatography–mass spectrometry (LC-MS), and a mass of 398.13278 (397.12257 m/z) was obtained. This shows that there is an antioxidant compound caulerpin in the sample extract.

#### Inhibitory Activity of Lipase (Enzymatic Assay)

*Lipase* was prepared using crude porcine pancreatic lipase (PPL) that was disintegrated in 50 mM phosphate buffer pH 7 (1 mg/ml) and centrifuged at 12,000 × *g* for 5 min to separate all insoluble. The enzyme stock concentration was set at about 0.1 mg/ml for every 1 mg of solid PPL dissolved in a 1 ml of buffer. *Lipase* activity was based on measuring the hydrolysis of p-nitrophenyl butyrate (pNPB) to p-nitrophenol using UV transparent microplate-96 at 405 nm on an ELISA reader. Lipase inhibition test was carried out by incubating sea grape extract (at concentrations of: 50, 100, 150, 200, 250 μg/ml) with PPL and pNPB in the reaction buffer (potassium phosphate buffer 50 mM, pH 7.2, 0.5% Triton X-100) for 10 min. pNPB was first put into 1% dimethyl sulfoxide (DMSO) for the final volume, after that diluted with a reaction buffer to a concentration of 2.5 mM in a 100 μL reaction. All assays were run at 37°C, and the reported results are the mean of the three replicates. Orlistat was used as a positive control. DMSO was utilized as negative control and its activity was observed with and without inhibitors. One unit of activity was established based on the rate of reaction yielding μmol p-nitrophenol per min at 37°C. *Lipase* inhibition activity was stated based on the activity percentage reduction when PPL was incubated in the test mixture. To ensure the validity of the data results, each sample was checked three times. Inhibition of *lipase* was expressed as a percentage and determined according to the formula below:


Lipase⁢inhibition⁢(I%)= 100-[(B-b)/⁢(A-a)×100]

where, A, activity without inhibitor; a, negative control without inhibitor; B, activity with inhibitor; B, negative control with inhibitor.

#### Inhibitory Activity of *α-Glucosidase* (Enzymatic Assay)

The *α-glucosidase* (Sigma-Aldrich; 1.52 UI/ml) was obtained by mixing 1 mg of powder (76 UI) with 50 ml of phosphate buffer (pH 6.9) received. The solution was then stored at 20°C. Sea grape extract with a concentration gradient of 0.1 ml (3 mg/ml) was then mixed with 0.35 ml of sucrose (65 mM) and maltose solution (65 mM). After heating (37°C, 5 min), 0.2 ml of *α-glucosidase* solution was added to the preheated system and then reacted at 37°C for 15 min. The reaction was carried out by heating the system in a water bath at 100°C. for 2 min. In this experiment, acarbose was used as a positive control. The control treatment (acarbose) was the same as the sea grape extract treatment. The *α-glucosidase* activity was expressed as the level of glucose production in the experiment. A total of 0.2 ml of the test solution was combined with the solution obtained in the *α-glucosidase* inhibition test, and then 3 ml of the color reagent was added to the reaction system. The system was then heated to 37°C for 5 min, and the absorbance of the solution was measured at a wavelength of 505 nm spectrophotometer (UV-VIS AMV11).

#### Inhibitory Activity of *α-Amylase* (Enzymatic Assay)

A 500-μl diluted sea grape extract sample, 500 μl 0.02 M sodium phosphate buffer (pH 6.9, 0.006 M NaCl), and 0.5 mg/ml porcine pancreatic amylase (effective concentration 3.2.1.1) were incubated at 25°C for 10 min. Furthermore, 500 μl of 1% strong starch solution in 0.02 M sodium phosphate buffer (pH 6.9; 0.006 M NaCl) was added to each mixture. The mixture was incubated for 10 min (25°C) and quenched with 1.0 ml of 3,5-dinitrosalicylic acid (C_7_H_4_N_2_O_7_) coloring reagent. The mixture was incubated in a water bath at 100°C for 5 min and allowed to stand at room temperature. The mixture was then diluted by adding 10 ml of distilled water, and the absorbance was measured at a wavelength of 540 nm at a spectrophotometer (UV-VIS AMV11). The reference sample contains all reagents and enzymes except the sample. Acarbose (Sigma-Aldrich) was used as a positive control.

### Clinical Trial

#### Dose Conversion

The best and most significant dose in a preliminary study (preclinical) in *Rattus norvegicus* rats is 150 mg/kg BW (body weight) (25). Afterward, the doses in this clinical trial study applied the table “Evaluation of Drug Activities: Pharmacometrics, ed. by D.R. Laurence and A.L. Bacharach” (28). The conversion factor from rat to human was 56.0. However, the dose used in the preliminary study is still within mg/kg BW while the conversion factor for rats must be per 200 g BW, so 150 is converted into 30 mg/200 g BW by dividing it by 5. Therefore, human dose = rat dose × conversion factor = 30 × 56.0 = 1,680 mg/70 kg BW or 1.68 g/70 kg BW.

#### Randomized-Double Blind Controlled Clinical Trial Protocol Design

The study was a 4-week, randomized, double-blind, placebo-controlled clinical trial followed by a 1-week screening period. Participants who respond to the invitation and meet the entry criteria during the telephone screening interview are scheduled for the initial visit. Evaluation during the initial visit includes physical examination in the form of body mass index (BMI) based on Asia-Pacific guidelines and blood parameter screening tests in the form of BG, triglycerides (TG), high-density lipoprotein (HDL), low-density lipoprotein (LDL), and TC, and PGC-1α was performed on all participants within 1 week of the initial screening. A random number between 1 and 70 was generated for each subject, and the registered participants were scheduled for their first visit and randomly assigned to the sea grape extract group (*n* = 35; Group B) or placebo (*n* = 35; Group A). Sea grape extract/placebo tablets/capsules were given to participants every week (1 day of consumption per oral; 1 capsule) 15 min before eating (according to the diabetes drug guideline consumption).

During the 4-week intervention period, participants were asked to continue their usual diet and not consume other dietary supplements. Anthropometric measurement of BMI (including both body weight and height) was measured using bioelectrical impedance analysis (BIA) (BC 541, Tanita Europe BV) with minimum clothing (subjects do not wear clothes and underwear) and measured by a nutritionist in a closed room for privacy. Measurements of waist circumference and hip circumference were done using a body tape scale (Medline MDR440FD) after anthropometric measurements to obtain the waist-hip ratio (WHR; waist circumference divided by hip circumference). Anthropometric (BMI and WHR), blood parameters, urine profile (a marker of toxicity), and nutrient intake of both groups were measured in the morning before and after the intervention period. During the trial phase, all participants were instructed to maintain their normal diet and physical activity (PA). Each week, participants were asked to report side effects or changes in training, lifestyle, or diet and to assess tablet compliance.

#### Subjects/Participants

Study participants were recruited in 2021 in Manado City, North Sulawesi, Republic of Indonesia. A total of 150 participants agreed to participate in the study. Only men who are obese (BMI ≥ 25 kg.m^–2^ and WHR ≥ 0.90) according to the Asia-Pacific guidelines (29) and have not been diagnosed with other diseases were included in the study. To meet the guidelines for evaluating the efficacy of functional foods referring to the Korea Food and Drug Administration (because Indonesia does not yet exist and Korea belongs to one Asian region used) (30), very obese participants (BMI <30 kg.m^–2^) were not included in the study.

Seventy participants met the research criteria with age 19–60 years old and were randomly divided into two groups (*n* = 35 each) given sea grape extract 1.68 g/70 kg BW day^–1^ (Group B) or placebo (1.68 g/70 kg BW day^–1^) (Group A). The exclusion criteria for this study are as follows: (a) significant weight variation (over 10%) in the last 3 months; (b) a history of cardiovascular disease including arrhythmia, heart failure or myocardial infarction, diabetes mellitus (DM) and the use of pacemakers; (c) a history of conditions that may interfere with test products or inhibit their absorption such as gastrointestinal diseases (Crohn’s disease) or surgeries that have been experienced (cesarean section or enterocele); (d) participation in other clinical trials in the last 2 months; (e) abnormal liver function; (f) a history of kidney disease (e.g., acute or chronic renal failure and nephrotic syndrome); (g) undergo antipsychotic drug therapy within the last 2 months; (h) laboratory test results, as well as medical or psychological conditions that may interfere with successful participation in research assessed by researchers; (i) a history of alcohol or substance abuse; (j) allergy or hypersensitivity to any of the ingredients in the test product; and (k) is neither a passive nor active smoker. All participants gave their written consent (assign informed consent) before the investigation begins. This research protocol has received ethical approval from the General Hospital Education Prof. Dr. R. D. Kandou, No. 142/EC/KEPK-KANDOU/VIII/2021 and registered with ClinicalTrials.gov, NCT05037591. All protocols referred to the Declaration of Helsinki and the Council for International Organizations of Medical Sciences (CIOMS).

#### Measurement of Clinical Outcome

A total of 70 participants who met the research criteria were asked to visit the clinic once every week (zeroth, first, second, third, and fourth/last week of the study period) with a total of five clinical visits, including an initial examination. During each visit, the use of the supplement is currently reviewed, and symptoms or side effects are noted. During screening visits, demographic and lifestyle information (age, alcohol consumption, and smoking habits) and medical history are noted, and urine turbidity tests are carried out.

##### Primary Outcome Measures

1. Peroxisome proliferator-activated receptor (PPAR)-γ coactivator 1 alpha levels “change” is being assessed. Time frame: change from baseline PGC-1α levels at the fourth week of the intervention period (evaluate PGC-1α level in pg/dL).
2. Total cholesterol “change” is being assessed (time frame: change from baseline TC at the fourth week of the intervention period). Evaluate TC level in mg/dL.
3. Blood glucose “change” is being assessed (time frame: change from baseline BG at the fourth week of the intervention period). Evaluate the total blood sugar level in mg/dL.

##### Secondary Outcome Measures

1. Body mass index (BMI) (time frame: change from baseline BMI at fourth week of the intervention period). BMI weight and height will be combined to report BMI in kg/m^2^.
2. Triglycerides (time frame: change from baseline triglycerides levels at fourth week of the intervention period). TG in mg/dL.
3. High-density lipoprotein (time frame: change from baseline HDL levels at fourth week of the intervention period). HDL in mg/dL.
4. Low-density lipoprotein (time frame: change from baseline LDL levels at fourth week of the intervention period). LDL in mg/dL.
5. Body weight (time frame: change from baseline body weight at the fourth week of the intervention period). Weight in kilograms (kg).
6. Body height (time frame: change from baseline body height at fourth week of the intervention period). Height in meters (m).
7. Waist-to-hip ratio (WHR) (time frame: change from baseline waist-to-hip ratio (WHR) at fourth week of the intervention period). This is calculated as waist measurement divided by hip measurement (W/H). For example, a person with a 30” (76 cm) waist and 38” (97 cm) hips has a WHR of about 0.78.

The following parameters are assessed: weight, height, WHR, and BMI during each visit. Blood samples were collected after a minimum of 12 h of fasting during the initial screening, as well as in the zeroth, first, second, third, and fourth weeks of the intervention period to obtain BG, TC, TG, HDL, LDL, and PGC-1α. Blood samples were taken from the arm vein. The sample is then centrifuged for 20 min at a speed of 3,000 rpm. Finally, the serum is collected for analysis of BG, TC, TG, HDL, LDL, and PGC-1α. Blood glucose, TG, HDL, LDL, and cholesterol levels were tested using the COBAS Integra^®^ 400 plus analyzer (Roche). The sample was washed with phosphate-buffered saline (PBS, pH 7.4) 1% until the liquid was clear. The sample was centrifuged at 3,000 rpm for 20 min to obtain pellets and supernatants. The supernatant is taken for PGC-1α examination. The concentration of PGC-1α is measured using the Human PGC-1α (PPARGC1A) (NM_013261) Untagged Human Clone Kit.

#### Evaluation of Supplementation Safety and Diet

The safety of the extract is assessed by the following procedure. Urine test strips with 10 verifiable parameters were used. Parameters examined included: glucose (50–100 mg/dl); protein (7.5–15 mg/dl); pH (5–9); leukocytes (9–15 leu/μl); nitrite (0.05–0.1 mg/dl); urobilinogen (0.2–1.0 mg/dl); blood (5–10 Ery/μl); ketones (2.5–5 mg/dl); bilirubin (0.4–1.0 mg/dl); and specific gravity (SG) (11,000–1,030). The above-mentioned urine parameters were tested using *Urinalysis 10U Reagent Strips Verify* (REF U031-102 exp. 05/03/2022). Pulse rate and blood pressure are measured on each visit after a 5-min break using the intelliVue MP70 (Philips, Netherlands). Personal reports are also recorded at these times. We kept the subjects maintaining their usual diet and activity, and all participants completed diet records on each visit to the clinic during the intervention period to evaluate their energy intake and the quality of their diet. Food and water intake data were collected using a Semiquantitative Food Frequency Questionnaire (SQ-FFQ) weekly, the Pittsburgh Sleep Quality Index (PSQI), and the Global Physical Activity Questionnaire (GPAQ) were analyzed by a nutritionist. The SQ-FFQ refers to the Food and Agriculture Organization (FAO) while the GPAQ refers to WHO. The Pittsburgh Sleep Quality Index (PSQI) is a sleep quality calculation with a value of 5 or higher indicating poor sleep quality. The higher the score, the worse the quality (31). The degree of adherence to taking sea grape tablets was measured using the pill count method referring to Rudd et al. (32) and Lam and Freco (33), and performed by a certified doctor. Moreover, subjects or participants were given pill boxes and medication reminder charts as a tool to remember to take medications.

#### Statistical Analyses and Justification of Randomized-Double Blind Controlled Clinical Trial Sample Size

Statistical analysis is performed using SPSS software, version 26 (IBM Corporation). Fixed effects include treatment groups, treatment visits, and interactions between treatment and visit groups. To determine the significance value of the outcome of 0 week or baseline characteristics and 0 week when compared with 4-week characteristics, the independent-sample *t-*test was used. P-trend from 0-week to 4-week intervention was analyzed using paired sample test (dependent sample *t*-test). Food, water intake, and PA were analyzed using one-way ANOVA. A suitable sample size for each group of studies was determined to be 32 participants/subjects, allowing a dropout rate of 20% [type I error is 5% (α = 0.05; Zα = 1.96), type II error is 20% (β = 0.2; Zβ = 0.842)] (34, 35). The *p* < 0.05 is considered statistically significant (CI 95%, two-sided with 80% power), and the data are presented as mean ± standard error of mean (SEM) for detecting the difference in body weight of 0.3 kg (SD or standard deviation = 0.60 kg) between the groups (34). Graphs were processed or created (Figures 3, 5) using GraphPad Prism Premium Version 9 for MacBook (GraphPad Software, LLC). The illustration in Figures 4, 6 uses a premium licensed Biorender belonging to one of the authors. The video abstract was created using Adobe Illustrator CS6 and Adobe After Effects 2021 with copyright-free vectors.

**FIGURE 2:**
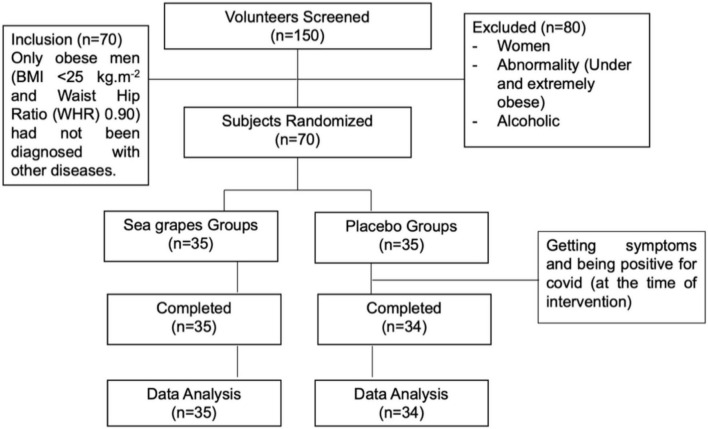
CONSORT diagram showing the flow of the study subject through the 4-week intervention.

**FIGURE 3:**
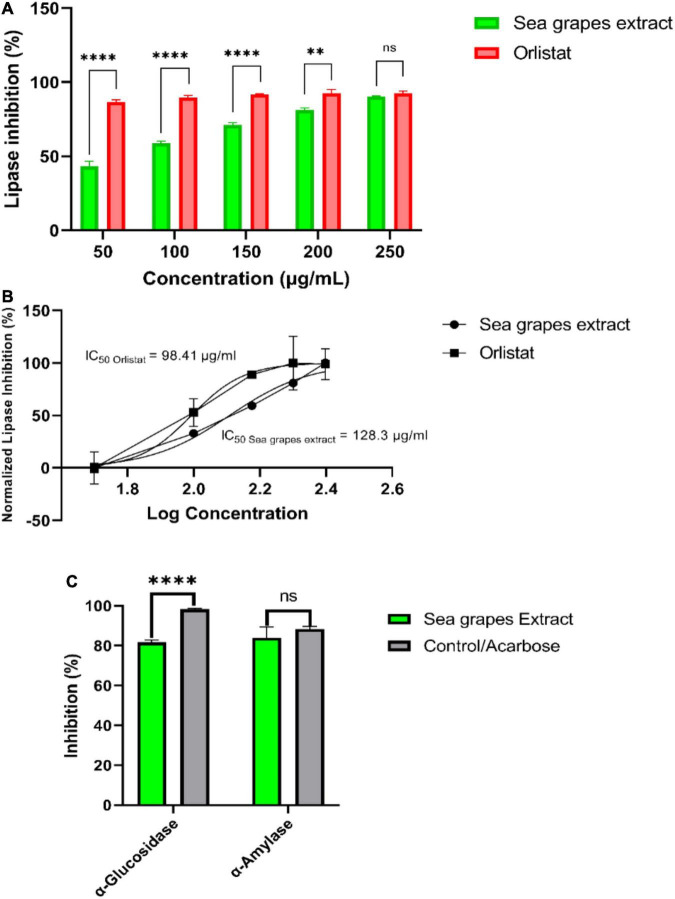
*In vitro* studies of sea grape inhibitory activity of *lipase, α-glucosidase*, and *α-amylase*. **p = 0.026; ****p < 0.0001.

**FIGURE 4:**
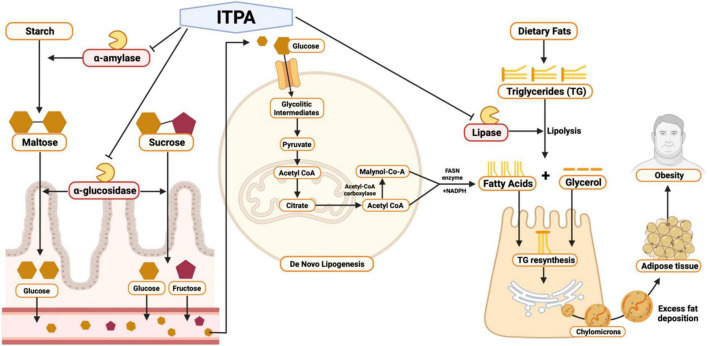
Pharmacological mechanism of sea grapes (*Caulerpa racemosa*) extract by *in silico* and *in vitro* studies.

**FIGURE 5:**
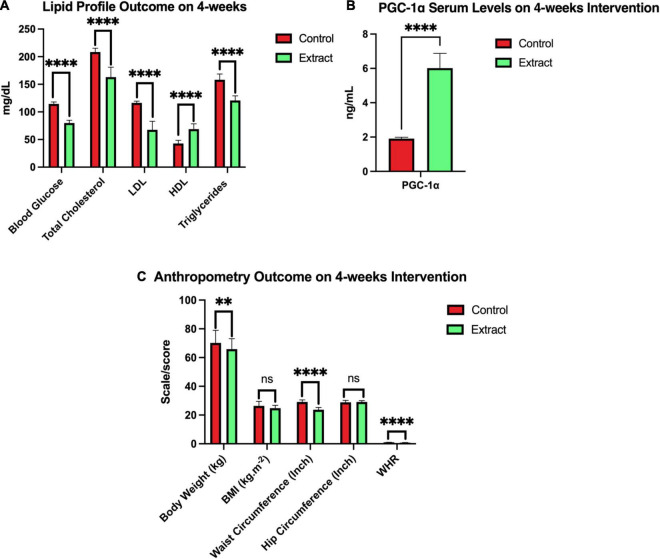
Outcome after 4 weeks of sea grapes extract supplementation. In this figure, there were three groups **(A-C)**. Group **A** showed a significant value of the lipid profile (blood glucose, total cholesterol, LDL, HDL, and triglycerides) between placebo (control) groups and extract (sea grapes antioxidant). Group **B** showed a significant value over the control group with the extract group 4 weeks of the intervention. Group **C** showed a significant value of the anthropometry profile at week 4 of the intervention between placebo or control groups and extract. *^ns^p* > 0.05; ^**^p = 0.026; ^****^p < 0.0001. The above values are presented in the form of mean ± SEM.

**FIGURE 6:**
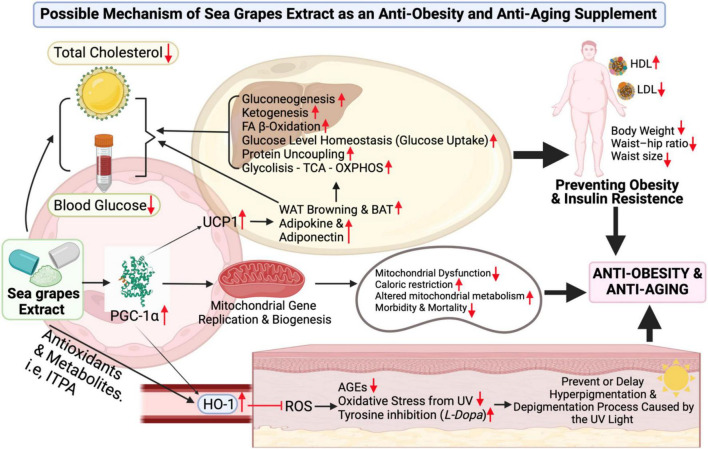
Sea grape extract effect possible-scheme for anti-obesity and anti-aging. Figure legends details directly can be found in the discussion. Abbreviations can be found at the top or title page.

## Results

### Metabolomic Assay

The metabolites profile of *C. racemosa* has been identified *via* an untargeted metabolomic profiling approach. There were six major compounds with mzCloudMS/MSlibrary values >75% (Table 1 and Supplementary 2).

### Molecular Docking Simulation

Major compounds that were successfully identified were continued *in silico* or molecular docking assay on *lipase, α-glucosidase*, and *α-amylase* enzymes computationally and validated, as listed in Table 2.

After validation, molecular docking tests on *lipase, α-glucosidase*, and *α-amylase* enzymes were performed on each major compound, acarbose (control for *α-glucosidase* and *α-amylase)* and orlistat (for *lipase).* It was found that 2-(1H-indol-3-yl)-3-[4-(trifluoromethyl)phenyl]acrylonitrile (ITPA) has the best result based on the ligand test in each receptor when compared with other compounds and controls. This comparison is based on the value of ΔG (kcal/mol), and the results of molecular docking tests are listed in Table 3.

**TABLE 3:** Molecular docking parameter of *C. racemosa* major compounds.

No.	Substance	Number in cluster (/100)			ΔG (kcal/mol)			Ki		
		1LPB	2QV4	3L4Y	1LPB	2QV4	3L4Y	1LPB	2QV4	3L4Y
1	Orlistat	5			−2.42			5.44 mM		
2	Acarbose		13	13		−4.22	−1.01		38.46 μM	3.76 mM
3	ITPA	62	73	71	−8.10	−6.65	−7.04	983.08 nM	11.03 μM	5.36 μM
4	DPA	92	39	71	−5.57	−4.77	−5.39	58.80 μM	204.49 μM	68.66 μM
5	BGPA	33	21	10	−4.68	−3.11	−1.61	21.17 μM	13.51 mM	15.43 mM
6	Betaine	98	27	97	−3.20	−3.97	−3.13	3.55 mM	447.08 μM	4.11 mM
7	Choline	95	100	60	−3.45	−3.90	−5.54	2.13 mM	1.20 mM	71.36 μM
8	Dibutyl phthalate	55	61	33	−6.22	−2.61	−4.35	12.63 μM	9.72 mM	251.95 μM

The best-docked compound 2-(1H-indol-3-yl)-3-[4-(trifluoromethyl)phenyl]acrylonitrile (ITPA) followed by the visualization of its amino acid interactions against human pancreatic *lipase*, *α-amylase*, and *α-glucosidase* is shown Table 4.

**TABLE 4:** Amino acid interaction of *C. racemosa* active compounds against human pancreatic *lipase*, *α-amylase*, and *α-glucosidase*.

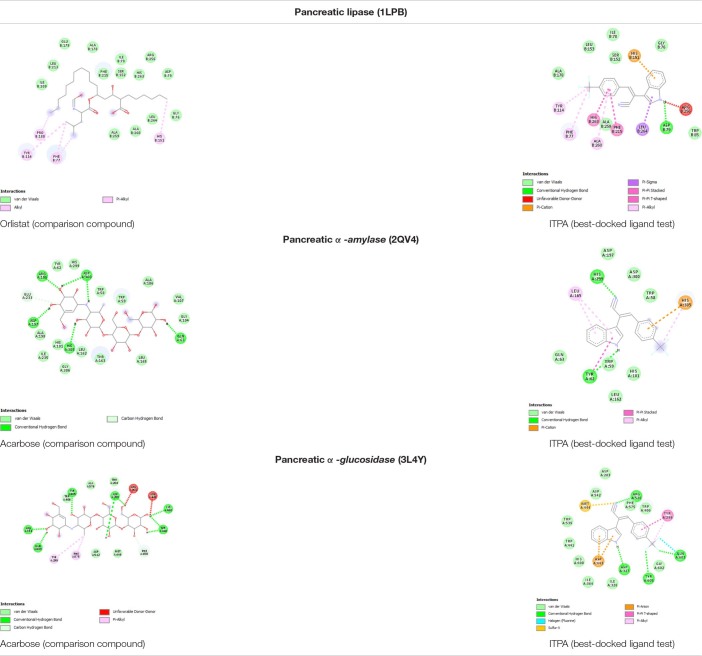

### Similarity of Sea Grapes by *in vitro* Inhibition With Acarbose and Orlistat

Figure 3 shows the result of *in vitro* studies. *Lipase* inhibition activity was compared between sea grape extract and orlistat (Figure 3A). Sea grape extract showed lower activities of *lipase* inhibition at doses of 50, 100, 150, and 200 μg/ml when compared with orlistat (*p* < 0.05). The *lipase* inhibition was similar between sea grape extract and orlistat at a dose of 250 μg/ml with a percentage of 90.30 ± 0.40 and 92.05 ± 1.54%, respectively. As shown in Figure 3, sea grape extract and orlistat yielded IC_50_ of 128.30 and 98.41 μg/ml, respectively (Figure 3B). *α-Glucosidase and α-amylase* inhibition activity was compared between sea grape extract and acarbose (Figure 3C). Sea grape extract showed lower activities of *α-glucosidase* (81.67 ± 1.54%) inhibition activity when compared with acarbose (98.25 ± 0.53%) (*p* < 0.05). In line with *lipase* inhibition of sea grape extract and orlistat at a dose of 250, the *α-Amylase* inhibition was similar between sea grape extract and acarbose with a percentage of 84.07 ± 5.28 and 88.33 ± 1.27%, respectively.

### Randomized-Double Blind Controlled Clinical Trial Study Participants

Among the 150 participants screened, 80 were excluded due to anthropometric characteristics, gender, and laboratory test results that did not meet the inclusion criteria. The remaining 70 participants met the study criteria and were divided equally into the sea grape extract group and the placebo group. In total, 70 participants met the research criteria (age 29.98 ± 3.26 years; weight, 71.28 ± 8.63 kg; BMI, 26.78 ± 1.44 kg.m^–2^). A participant in the placebo group had to be excluded from the study because of having symptoms and being positive for COVID-19 (at the time of the intervention). In the end, 69 participants [35 extracts of sea grapes (Group B) and 34 participants of the placebo (Group A)] were able to complete the study and used in the analysis of the study results (Figure 2).

### Baseline Participant Characteristics

The general characteristics of the participants are listed in Table 5 (Baseline/0-weeks intervention). There were no significant differences (independent-sample *t*-test CI 95%) in early anthropometric characteristics such as height, weight, waist circumference, hip circumference, WHR, and BMI and laboratory test results that included BG, TC, PGC-1α, LDL, HDL, and TG, as well as in PA and Pittsburgh Sleep Quality Index (PSQI) scores between the sea grape extract group (B) and placebo (A). However, at age, there is a significant difference (*p* < 0.05). Although there are significant differences, the upper age limit (21–36 years) is still in the adult category. Independent *t*-tests were also conducted for food and water intake based on Semiquantitative Food Frequency Questionnaire (SQ-FFQ) data, and there was no difference between the sea grape extract group and placebo in the baseline. Urine levels are all the same so they cannot be computed by SPSS [urine is all normal (100%)] (Supplement Table 5).

**TABLE 5:** Characteristics of the participants on the baseline (0 week).

Parameters	Placebo	Extract	*P*-value*
Age (years)	29.18 ± 3.91	30.77 ± 2.27	0.042
Body mass index (BMI; kg.m^–2^)	26.92 ± 1.56	26.64 ± 1.31	0.413
Waist circumference (inch)	29.13 ± 1.09	28.92 ± 1.03	0.423
Hip circumference (inch)	28.9 ± 1.06	29.08 ± 1.02	0.478
Waist-hip ratio (WHR)	1.00 ± 0.04	0.99 ± 0.04	0.185
PGC-1α (ng/mL)	1.83 ± 0.10	1.87 ± 0.10	0.071
Blood glucose (mg/dL)	113.36 ± 5.89	113.68 ± 6.56	0.831
Total cholesterol (mg/dL)	212.55 ± 8.65	213.41 ± 8.27	0.674
High-density lipoprotein (HDL; mg/dL)	44.08 ± 5.31	42.61 ± 4.92	0.236
Low-density lipoprotein (LDL; mg/dL)	120.25 ± 8.64	118.78 ± 7.66	0.455
Triglycerides (mg/dL)	160.45 ± 9.74	158.17 ± 8.92	0.313
Food intake (Kcal)	2341.57 ± 0.50	2341.79 ± 0.48	0.062
Water intake (L)	3.40 ± 0.00	3.40 ± 0.00	1.000

*The significance value of the results of the independent sample t-test data analysis (at 95% CI). The above values are presented in the form of mean ± SEM.

In the placebo group (Group A), participants who had strenuous PA were four people (57.10%), and in the sea grape extract group (Group B), there were three people (42.90%), while there were 30 people (48.40%) and 32 people (51.60%) in Group A and Group B who had moderate PA (Supplementary Table 1). In the placebo group (Group A), 28 participants had a good PSQI score (48.30%) and six participants had a poor PSQI score (54.50%); while in the sea grape extract group (Group B), there were 30 people (51.70%) with a good PSQI score and five people (45.50%) with a poor PSQI score (Supplementary Table 1).

The sea grape extract group showed a pattern of significant decrease from 0 week or before the intervention to 4 weeks in the parameters of BG, TC, LDL, TG, body weight, waist circumference, and WHR (*p* < 0.05) (Table 6); even though the BMI parameter at week 4 between the control and extract groups did not show a significant difference (Figure 5). There was also a significant increase in serum PGC-1α and high-density lipoprotein (*p* = 0.000) in subjects treated with sea grape extract.

**TABLE 6:** P-trend from 0-week to 4-week intervention.

Parameters	Groups	0-week/Baseline	4-weeks	*P*-value
*Blood glucose (mg/dL)*	Placebo	113.36 ± 5.89	114.49 ± 3.46	0.306
	Extract	113.68 ± 6.56	79.82 ± 4.99	0.000*
*Total cholesterol (mg/dL)*	Placebo	212.55 ± 8.65	208.36 ± 7.04	0.050
	Extract	213.41 ± 8.27	162.94 ± 18.07	0.000*
*PGC-1α (ng/mL)*	Placebo	1.83 ± 0.10	1.91 ± 0.07	0.000*
	Extract	1.87 ± 0.10	6.01 ± 0.85	0.000*
*High-density lipoprotein (HDL; mg/dL)*	Placebo	44.08 ± 5.31	42.78 ± 5.86	0.366
	Extract	42.61 ± 4.92	68.83 ± 9.60	0.000*
*Low-density lipoprotein (LDL; mg/dL)*	Placebo	120.25 ± 8.64	116.26 ± 3.13	0.021*
	Extract	118.78 ± 7.66	67.66 ± 15.36	0.000*
*Triglycerides (mg/dL)*	Placebo	160.45 ± 9.74	158.22 ± 10.49	0.201
	Extract	158.17 ± 8.92	120.64 ± 8.45	0.000*
*Body* m*ass* i*ndex (BMI; kg.m^–2^)*	Placebo	26.92 ± 1.56	26.40 ± 3.12	0.209
	Extract	26.64 ± 1.31	24.76 ± 1.98	0.000*
*Body* w*eight (kg)*	Placebo	71.99 ± 9.54	70.24 ± 8.74	0.251
	Extract	70.59 ± 7.71	65.85 ± 7.27	0.000*
*Waist circumference (inch)*	Placebo	29.13 ± 1.09	29.13 ± 1.36	1.000
	Extract	28.92 ± 1.03	23.74 ± 1.57	0.000*
*Hip circumference (inch)*	Placebo	28.9 ± 1.06	28.86 ± 1.31	0.840
	Extract	29.08 ± 1.02	29.08 ± 0.97	0.976
*Waist-hip ratio (WHR)*	Placebo	1.00 ± 0.04	1.01 ± 0.06	0.805
	Extract	0.99 ± 0.04	0.81 ± 0.05	0.000*

*Significant (p < 0.05) from the results of data analysis of the dependent sample t-test or paired sample test (95% CI). The above values are presented in the form of mean ± SEM.

After 4 weeks of intervention, there was a significant decrease (*p* < 0.05) in BG, TC, LDL, TG, waist circumference, WHR (*p* = 0.000), and body weight (*p* = 0.026). There was also a significant increase (*p* = 0.000) in PGC-1α and HDL, respectively, in the sea grape extract group when compared with the placebo group (Figure 5). The independent *t*-test of BMI between groups A and B in the fourth week of intervention showed that there was no significant difference (*p* > 0.05) (Figure 5). However, in group B (sea grape extract), there was a decrease in BMI from the baseline after 4 weeks of intervention.

Based on the results of the one-way ANOVA statistical test, there were no differences in the intake of food, drink (water), and physical activity ranging from 0 to 4 weeks of intervention (Table 7).

**TABLE 7:** Food and water intake, and physical activity (PA) during intervention (0–4 weeks).

Parameters	0-week/Baseline	1-week	2-weeks	3-weeks	4-weeks	*P*-value
***Placebo group***						
Food intake (Kcal)	2,341.46 ± 0.41	2,341.42 ± 0.07	2,341.45 ± 0.11	2,341.49 ± 0.70	2,341.40 ± 0.28	0.457
Water intake (L)	3.40 ± 0.03	3.40 ± 0.03	3.41 ± 0.02	3.41 ± 0.29	3.41 ± 0.05	0.238
Physical activity	1.12 ± 0.32	1.12 ± 0.32	1.12 ± 0.32	1.12 ± 0.32	1.12 ± 0.32	1.000*
***Extract group***						
Food intake (Kcal)	2,341.94 ± 0.46	2,341.94 ± 0.40	2,342.03 ± 0.61	2,342.07 ± 0.34	2,341.85 ± 0.37	0.062
Water intake (L)	3.40 ± 0.29	3.41 ± 0.05	3.41 ± 0.03	3.40 ± 0.01	3.40 ± 0.00	0.544
Physical activity	1.09 ± 0.28	1.09 ± 0.28	1.09 ± 0.28	1.09 ± 0.28	1.09 ± 0.28	1.000*

*The score that shows the most that physical activity is in the moderate category; with a value near 0 is categorized as low physical activity; 1 is moderate, and 2 is a high physical activity level. The above values are presented in the form of mean ± SEM.

This indicates that the participants adhered to the protocol of not changing their diet and activities (the same as in 0-week/baseline). There was no clinically significant change (urine indicator) in any of the safety parameters observed. Therefore, this extract supplementation is safe to consume.

## Discussion

Indonesia’s marine potential is not only rich in biodiversity but also has a myriad of benefits that can be used for the health of mankind (36). Sea grapes or *C. racemosa* have many benefits, antioxidants, and other bioactive compounds that are undoubtedly beneficial (19, 20, 22, 27, 37). One of the typical compounds that serve as an antioxidant is Caulerpin (27). Several molecular coupling studies mapped out its potential benefits for a variety of noncommunicable diseases, such as cancer, diabetes, etc. (38–40). In this study, we also succeeded in profiling secondary metabolic compounds from the sea grape extract through metabolomic profiling LC-HRMS (Supplementary 2). There are six major compounds contained in the ethanolic extract, such as 3-[3-(beta-D-glucopyranosyloxy)-2-hydroxyphenyl]propanoic acid (BGPA), 2-(1H-indol-3-yl)-3-[4-(trifluoromethyl)phenyl]acrylonitrile (ITPA), 2-(3,4-dihydroxyphenyl)acetamide (DPA), betaine, choline, and dibutyl phthalate (Table 1). The success of this identification adds value to the novelty and source profile of compounds contained in sea grape extract and is useful for other researchers in developing products based on sea grape extract. Of course, these compounds are strongly suspected to contribute to improving lipid profiles and PGC-1α in this clinical study.

Based on molecular docking simulation, the docking protocol was valid as shown by the RMSD <2.0 angstrom (Table 2). Out of six major compounds found in *C. racemosa*, ITPA performed as the best dock ligand that can interact with human pancreatic lipase, *α-glucosidase*, and *α-amylase* through multi-targeted activity. ITPA has the lowest binding energy against lipase (ΔG = −8.10 kcal/mol) while the FDA-approved drug (orlistat, comparison compound) has −2.42 kcal/mol of free binding energy. On the other hand, ITPA also has good inhibitory activity against *α-amylase* (ΔG = −6.65 kcal/mol), the lowest among all testing ligands. *α-Glucosidase* also has been inhibited by ITPA with a strong binding affinity (ΔG = −7.04 kcal/mol) (Table 3). These results conclude that ITPA is predicted to be a substance of *C. racemosa* extract that is responsible for the anti-obesity activity.

The pharmacological mechanism of *C. racemosa* extract *in silico* and *in vitro* studies is shown in Figure 4. In the carbohydrate digestion process, starch that enters the digestive tract is cleaved by pancreatic and salivary α-amylase at its α-(1–4) glycosidic bonds, forming disaccharides such as maltose (41). When passing the small intestines, α-glucosidase which is located on the brush borders catalyzes the hydrolysis of disaccharide linkages, breaking them down to monosaccharides, such as glucose, that are then further transferred to the bloodstream (42). After consuming high carbohydrate and high-fat diets, *de novo* lipogenesis which is the conversion of glucose into a fatty acid (FA) in the liver and adipose tissues can be further triggered (43). *De novo* lipogenesis starts with glucose cellular uptake, in which then glucose is phosphorylated to glycolytic intermediates such as glucose-6-phosphate, which are subsequently processed into pyruvate and ATP. Inside the mitochondria, pyruvate is then transformed into acetyl CoA, going into the citric acid cycle. Citrate is then transported into the cytosol to be converted back to acetyl CoA by ATP citrate lyase. Next, carboxylation of acetyl CoA by acetyl CoA carboxylase occurs, forming malonyl CoA. Both acetyl CoA and malonyl CoA are then converted to a FA by fatty acid synthase (FASN) with the aid of NADPH in the cytosol (44).

Interestingly, sea grape extract also showed anti-obesity potential seen by its *lipase* inhibition activity *in vitro*, as suggested by *in silico studies* (Figure 3A and Table 4). The *lipase* inhibitory activity of sea grape extract was similar to orlistat at a dose of 250 μg/ml (Figure 3B). In lipid metabolism, triglycerides from dietary fats are broken down through lipolysis to monoglycerides and FAs by mainly pancreatic and gastric lipase (Figure 4). Monoglycerides and FAs are then transported into epithelial cells in the mucous membrane of the small intestine. These are then converted back to triacylglycerol at the expense of ATP (45). The newly formed triacylglycerol is subsequently combined with apolipoproteins, forming chylomicrons. Chylomicrons transport absorbed lipid to body tissues, especially adipose tissue, excess fat deposition will cause obesity (45). In addition, sea grape extract also showed anti-obesity and anti-aging *via* antidiabetic potential seen by its *α-glucosidase* and *α-amylase* inhibition activity, as suggested by *in silico studies* (Figure 3 and Table 4). The *α-glucosidase* inhibitory activity of the sea grape extract was 81.67 ± 1.54%. The *α-amylase* inhibitory activity was similar between sea grape extract and acarbose with a percentage of 84.07 ± 5.28 and 88.33 ± 1.27%, respectively.

Previous *in vivo* (preclinical study) studies showed that sea grape extract improves BG, TC, and serum PGC-1α in mice with a cholesterol- and fat-enriched diet (22, 25). Then, the opportunity was continued in this clinical trial, with the results showing alignment between the results in the previous trial animals with the results of this clinical trial. In this clinical trial, the investigators used obese men as subjects or participants because they considered hormonal factors in women since there is a dynamic of hormonal changes that can potentially result in bias in the study (46, 47). Furthermore, men are most vulnerable to central obesity and are at a higher risk of developing it compared to women. Harbuwono et al. supported this finding that men tend to be more susceptible to central obesity (1).

After 4 weeks of intervention, there was a significant decrease in BG, TC, LDL, TG, waist circumference, WHR, and body weight. There was also a significant increase in PGC-1α and HDL, respectively, in the sea grape extract group when compared with the placebo group (Figure 5). The one-way ANOVA test of PA, food, and water intake from 0 to 4 weeks showed no change (*p* > 0.05 which means controlled) between the groups presented in Table 7. It also shows that PA and food and water intake in study subjects did not become confounding variables.

A significant increase in PGC-1α has been observed from the results of this clinical trial. As Cheng et al. (48) have pointed out, PGC-1α is an important factor in the regulation of lipid and metabolic processes (Figure 6) (48). Its role ranges from increasing heme oxygenase-1 (HO-1), which plays a role in inhibiting the incidence of reactive oxygen species (ROS) (49) along with antioxidants contained in sea grape extract to reducing advanced glycation end-product (AGE), oxidative stress from ultraviolet (UV) light and contribute to increasing inhibition of enzyme tyrosinase (L-Dopa) (50). This leads to the prevention of hyperpigmentation and depigmentation caused by UV rays (Figure 6) (51). In addition, antioxidants also play a role in increasing collagen production and minimizing inflammatory events in the body if consumed (52). It is also known from other studies that PGC-1α and antioxidants play a role in neuroprotective against neurodegenerative disorders-related aging diseases (34, 53).

In Figure 6, it is explained that PGC-1α also has mechanisms to increase uncoupling protein 1 (UCP1) (54), UCP1 increases the browning process of white adipocyte tissue (WAT Browning) and increases brown adipocyte tissue (BAT), as well as an increase in adipokine (adiponectin) (48). PGC-1α–UCP-1 contributes to glucose uptake control (glucose homeostasis), glycolysis, -oxidation of FA β-oxidation, tricarboxylic acid cycle, oxidative phosphorylation (OXPHOS), mitochondrial biogenesis, gluconeogenesis, ketogenesis, and protein uncoupling (Figure 6) (48). So, it was in line with the administration of sea grape extract in this clinical trial (dose of 1.68 g/70 kg BW/day), lowering TC, BG, LDL, and TG in male subjects with obesity (significantly). It is also suspected to contribute strongly to weight loss, WHR, and waist size. The extract of sea grapes that increases PGC-1α serum will also lead to an increase in mitochondrial gene replication and biogenesis (11, 13, 48). Therefore, mitochondrial repair increases, caloric restriction increases, altered mitochondrial metabolism increases, and thus lowers the risk of morbidity and mortality (Figure 6) (48).

The facts that have been mentioned before support and show how sea grapes contribute as an anti-obesity and anti-aging supplement. However, it is necessary to continue the study for a longer period to see significant changes in BMI, which in this study did not show a significant effect in decreasing BMI after the administration of sea grape extract (*p* > 0.05), compared with the placebo or control groups. In line with research by Mohammadi et al. (55), the administration of curcuminoids for 30-days did not have a significant impact on the BMI of obese subjects. However, a study by Karandish et al. (56) with an intervention time of 90 days found that there was a significant reduction in BMI. Due to the limited research conducted for sea grape extract intervention and with no funding provided, this study was only 30 days (4 weeks) long. However, in Group B (sea grape extract), there was a decrease significantly (*p* = 0.000) in BMI from baseline to after 4 weeks of intervention (Table 6). It is also necessary to conduct clinical trials on diabetics to know the complex effect of an anti-noncommunicable disease. This clinical trial is the first human study conducted to examine the effects of sea grapes on health, especially obesity and anti-aging. The potential of this novelty needs to be further developed, such as the commercial implementation of products so that health effects can be adjusted and the impact in efforts to accelerate the decline in obesity. Based on the results of the pill count data collection by the doctor on duty, compliance of the consumption of sea grape tablets reached 100% and we did not analyze blood biomarkers/urine to confirm the compounds. However, this became a limitation of this study, given that this is the first human clinical trial conducted to see the efficacy of sea grapes. Subsequent clinical trials shall observe at blood biomarkers/urine markers to confirm the compounds, specifically. Moreover, the isolation, purification, and synthesis of each potential metabolite compounds which have been explored are urgently needed to prove sea grape (*C. racemosa*) extract as an anti-obesity and anti-aging agents, and if possible, assay it on human adipose tissues.

## Conclusion

Six major compounds were identified from sea grape (*C. racemosa*) extract; these compounds exhibit inhibition activity to *lipase*, *α-glucosidase*, and *α-amylase*, as shown by *in silico* and *in vitro* studies. A dose of 1.68 g/70 kg BW/day sea grape extract (Group B) has significant benefits in improving lipid profiles, waist circumference size, WHR, and body weight as well as improving serum PGC-1α in men with obesity. Our findings revealed that sea grape extract is a potent anti-obesity supplement that may not produce any significant adverse effects. These results suggest that sea grape extract supplementation may be effective in treating individuals with obesity as a functional anti-obesity and potential anti-aging food supplement.

## Patents

Patent number S00202107179 (FN is a patent holder, https://pdki-indonesia.dgip.go.id/detail/S00202107179?type=patent&keyword=S00202107179).

## Data Availability Statement

The raw data supporting the conclusions of this article will be made available by the authors, without undue reservation.

## Ethics Statement

The studies involving human participants were reviewed and approved by the General Hospital Education Prof. Dr. R.D.Kandou, No. 142/EC/KEPK-KANDOU/VIII/2021 and registered with ClinicalTrials.gov, NCT05037591 and indexed by International Clinical Trials Registry Platform World Health Organization (https://trialsearch.who.int/Trial2.aspx?TrialID=NCT05037591). The patients/participants provided their written informed consent to participate in this study.

## Author Contributions

FN, HP, NM, NS, FI, and HaH: designed the research, investigation, project administration, writing, and revising of the original draft. NT, NS, JV, DK, SF, TS, IK, HoH, and NM: supervision, validation, and writing–review and editing. MT, VY, RR, and MB: assisting in the processing of data on food intake, drinking, and physical activity as well as helping to revise and edit video abstract. All authors contributed to the article and approved the submitted version.

## Conflict of Interest

The authors declare that the research was conducted in the absence of any commercial or financial relationships that could be construed as a potential conflict of interest. The reviewer IN declared a past co-authorship with the author FN to the handling editor.

## Publisher’s Note

All claims expressed in this article are solely those of the authors and do not necessarily represent those of their affiliated organizations, or those of the publisher, the editors and the reviewers. Any product that may be evaluated in this article, or claim that may be made by its manufacturer, is not guaranteed or endorsed by the publisher.

## Supplementary Material

Supplementary 1 Crosstab physical activity (PA), PSQI, urine dan education.

Supplementary 2 Metabolomic profiling of sea grape extract.

Supplementary 3 Graphical video.

## Abbreviations

AGE: advanced glycation end-product
BAT: brown adipocyte tissue
BG: blood glucose
BMI: body mass index
DPPH: 2,2-diphenyl-1-picrylhydrazyl
FA: fatty acid
HDL: high-density lipoprotein
HO-1: heme oxygenase-1
LC-MS: liquid chromatography–mass spectrometry
LDL: low-density lipoprotein
OXPHOS: oxidative phosphorylation
PA: physical activity
PGC-1 α: peroxisome proliferator-activated receptor (PPAR)- γ coactivator 1 alpha
PSQI: Pittsburgh Sleep Quality Index
RCT: randomized-double blind controlled clinical trial
ROS: reactive oxygen species
SQ-FFQ: Semiquantitative Food Frequency Questionnaire
TC: total cholesterol
TG: triglycerides
UCP1: uncoupling protein 1
UV: ultraviolet
WAT: white adipocyte tissue
WHR: waist-hip ratio.
