# Supplementary material for: Metabolomic Assay, Computational Screening, and Pharmacological Evaluation of Caulerpa racemosa as an Anti-obesity With Anti-aging by Altering Lipid Profile and Peroxisome Proliferator-Activated Receptor-γ Coactivator 1-α Levels
Source: Front Nutr. 2022 Jul 14;9:939073. doi: 10.3389/fnut.2022.939073 (PMC9330592; doi:10.3389/fnut.2022.939073)
Supplement: Supplementary 1 — Crosstab physical activity (PA), PSQI, urine dan education. [file Data_Sheet_1.PDF]

Table Suplementary 1. Crosstab Physical Activity (PA), PSQI, Urine dan Education

Baseline/0 Week:

Crosstab

			PA		Total
			Moderate	Strenuous	
Groups	Placebo	Count	30	4	34
		% within PA	48.40%	57.10%	49.30%
	Extract	Count	32	3	35
		% within PA	51.60%	42.90%	50.70%
Total		Count	62	7	69
		% within PA	100.00%	100.00%	100.00%

In the placebo group, participants who had strenuous physical activity were 4 people (57.10%) and in the sea grape extract there were 3 people (42.90%).
While in the placebo group who had moderate physical activity as many as 30 (48.40%) and the extract group as many as 32 (51.60%)

Crosstab

			PSQI		Total
			Good	Poor	
Groups	Placebo	Count	28	6	34
		% within PSQI	48.30%	54.50%	49.30%
	Extract	Count	30	5	35
		% within PSQI	51.70%	45.50%	50.70%
Total		Count	58	11	69

		% within PSQI	100.00%	100.00%	100.00%

In the placebo group, 28 people (48.30%) had a good PSQI score and 30 people (51.70%) in sea grape extract (51.70%).
Meanwhile, in the placebo group, 6 (54.50%) had poor PSQI and 5 (45.50%) extract groups.

Crosstab

			Urine	Total
			Normal	
Groups	Placebo	Count	34	34
		% within Urine	49.30%	49.30%
	Extract	Count	35	35
		% within Urine	50.70%	50.70%
Total		Count	69	69
		% within Urine	100.00%	100.00%

Crosstab

			Education	Total
			Bachelor Degrees (Strata 1)	
Groups	Placebo	Count	34	34
		% within Education	49.30%	49.30%
	Extract	Count	35	35
		% within Education	50.70%	50.70%
Total		Count	69	69
		% within Education	100.00%	100.00%
