# Supplementary material for: Metabolomic Assay, Computational Screening, and Pharmacological Evaluation of Caulerpa racemosa as an Anti-obesity With Anti-aging by Altering Lipid Profile and Peroxisome Proliferator-Activated Receptor-γ Coactivator 1-α Levels
Source: Front Nutr. 2022 Jul 14;9:939073. doi: 10.3389/fnut.2022.939073 (PMC9330592; doi:10.3389/fnut.2022.939073)

LABORATORIUM SENTRAL ILMU HAYATI
UNIVERSITAS BRAWIJAYA

Jl. Veteran Malang 65145
Telp. (0341) 559054; Fax (0341) 559054; HP: 081 803 823727
http://lsih.brawijaya.ac.id
Email: labsentralub@ub.ac.id; labsentralub@gmail.com

Sample Name : MSRJ
Solvent : Etanol
Injection Method : Fullscreen Positive
Data : Best Match

Name	Formula	Calc. MW	RT [min]	Area (Max.)	mzCloud Best Match
3-[3-(beta-D-Glucopyranosyloxy)-2-hydroxyphenyl]propanoic acid	C15 H20 O9	366.0987	15.508	2,607,709,506.66	73.7
Choline	C5 H13 N O	103.0996	0.948	1,702,868,432.32	97.2
Betaine	C5 H11 N O2	117.0787	0.952	815,230,823.19	96.3
Dibutyl phthalate	C16 H22 O4	278.1504	18.019	530,234,031.11	99.2
2-(1H-indol-3-yl)-3-[4-(trifluoromethyl)phenyl]acrylonitrile	C18 H11 F3 N2	312.0885	10.763	484,746,646.13	76.1
2-(3,4-dihydroxyphenyl)acetamide	C8 H9 N O3	189.0429	0.931	464,700,110.40	84.5
2,2,6,6-Tetramethyl-1-piperidinol (TEMPO)	C9 H19 N O	157.146	12.259	312,358,366.23	93.5
Hexadecanamide	C16 H33 N O	255.2549	21.93	275,377,138.03	97.3
Diisobutylphthalate	C16 H22 O4	278.1504	17.905	273,288,427.76	98.5
Choline	C5 H13 N O	103.0996	25.068	270,900,405.44	97.9
Choline	C5 H13 N O	103.0996	22.959	215,421,162.79	97.6
5-(2-Thienyl)nicotinic acid	C10 H7 N O2 S	205.0169	0.927	209,833,574.73	95.5
Isoamylamine	C5 H13 N	87.10487	0.916	204,192,224.93	89.8
NP-008993	C18 H34 O4	296.2341	18.231	188,856,575.32	91.6
Palmitoleic Acid	C16 H30 O2	276.2077	15.894	188,602,459.59	65.1
Hexadecanamide	C16 H33 N O	255.2549	21.709	128,717,637.06	98.8
9-Oxo-10(E),12(E)-octadecadienoic acid	C18 H30 O3	294.2184	17.615	128,180,581.48	95.8
(±)13-HpODE	C18 H32 O4	294.2183	17.427	123,110,274.85	93.6
6-Gingerol	C17 H26 O4	294.1819	13.158	120,142,739.65	65.7
Adenine	C5 H5 N5	270.108	1.001	115,965,792.41	81.7
Oleamide	C18 H35 N O	281.2706	21.564	113,705,808.63	97.4
α-Eleostearic acid	C18 H30 O2	278.2234	16.815	101,832,983.44	94.4
NP-001596	C16 H30 O4	268.2027	16.451	95,661,314.03	75.9
4-{[(4,6-Dimethoxypyrimidin-2-yl)amino]methylidene}-2-phenyl-4,5-dihydro-1,3-oxazol-5-one	C16 H14 N4 O4	326.1044	11.582	85,055,308.35	63.1
α-Eleostearic acid	C18 H30 O2	278.2235	16.95	84,895,782.05	94.4
Cuminaldehyde	C10 H12 O	148.0883	14.177	82,274,117.71	89.7

1,2-dihydroxyheptadec-16-yn-4-yl acetate	C19 H34 O4	326.2445	15.669	79,749,057.14	77.6
Adenosine	C10 H13 N5 O4	267.0956	1.024	79,366,759.62	97.7
Diisobutylphthalate	C16 H22 O4	278.1504	17.729	76,134,032.37	98.5
α-Linolenic acid	C18 H30 O2	278.2234	20.046	75,757,786.32	97.4
ethyl 3-oxo-5,6-diphenyl-2,3-dihydropyridazine-4-carboxylate	C19 H16 N2 O3	342.0991	17.351	73,810,505.50	81
12-Oxo phytodienoic acid	C18 H28 O3	292.2026	16.633	70,444,302.73	88.1
Nicotinamide	C6 H6 N2 O	122.0476	1.044	70,264,037.81	83.9
Stearamide	C18 H37 N O	283.2858	23.794	66,634,752.38	96.8
ethyl 3-oxo-5,6-diphenyl-2,3-dihydropyridazine-4-carboxylate	C19 H16 N2 O3	342.0991	17.066	64,011,618.68	79.5
Oleoyl ethanolamide	C20 H39 N O2	307.2861	21.936	62,781,318.81	75.9
Choline	C5 H13 N O	103.0996	25.747	62,074,841.62	97.9
NP-020014	C15 H26 O3	276.1714	13.43	61,670,246.92	68.6
NP-008993	C18 H34 O4	296.2341	18.478	59,612,361.95	83.6
(±)11(12)-EET	C20 H32 O3	302.2233	17.56	58,853,299.74	92
Ouabain	C29 H44 O12	622.2406	20.518	57,317,714.92	82.6
1-(4-methoxyphenyl)propane-1,2-diol	C10 H14 O3	164.0831	12.847	56,359,590.26	78.7
Stearamide	C18 H37 N O	283.2858	23.208	53,830,529.61	97.6
4-Fluoro-α-pyrrolidinobutiophenone	C14 H18 F N O	235.1412	0.873	53,487,771.60	79.6
Pyridoxal	C8 H9 N O3	167.0611	1.305	53,461,055.41	93.7
(4aR,5R,6R)-6-hydroxy-4a,5-dimethyl-3-(prop-1-en-2-yl)-2,4a,5,6,7,8-hexahydronaphthalen-2-one	C15 H20 O2	214.135	14.862	53,396,851.21	68.3
Pyridoxal	C8 H9 N O3	167.0611	0.895	52,209,470.02	90.1
Ouabain	C29 H44 O12	622.2406	19.718	51,946,909.47	73.6
Choline	C5 H13 N O	103.0996	0.104	51,935,443.62	97.5
(3S,3aR,4S,4aR,7aR,8R,9aR)-3,4a,8-trimethyl-2,5-dioxo-2H,3H,3aH,4H,4aH,5H,7aH,8H,9H,9aH-azuleno[6,5-b]furan-4-yl 2-methylpropanoate	C19 H26 O5	356.1586	14.874	51,133,618.50	74.7
1-(4-chlorobenzyl)-2-{[(4-methylphenyl)thio]methyl}-1H-imidazole	C18 H17 Cl N2 S	328.0835	15.089	47,187,926.45	60.3
9-Oxo-10(E),12(E)-octadecadienoic acid	C18 H30 O3	294.2184	17.898	46,549,339.30	97.7
(+/-)11(12)-EET	C20 H32 O3	302.2233	17.114	46,373,202.63	94.9
Choline	C5 H13 N O	103.0996	20.056	45,416,702.95	97.5
1,4-dihydroxyheptadec-16-en-2-yl acetate	C19 H36 O4	328.2602	16.835	44,926,299.27	71.6
Methaqualone	C16 H14 N2 O	500.2191	22.189	43,991,117.28	83.7

Stearamide	C18 H37 N O	283.2858	22.313	42,372,869.06	93
OPEO	C16 H26 O2	250.1923	16.48	41,454,327.51	64.7
Palmitic Acid	C16 H32 O2	273.2655	14.714	38,806,980.21	84.3
(±)8-HEPE	C20 H30 O3	300.2076	16.334	37,032,281.51	92.1
(±)8-HEPE	C20 H30 O3	300.2076	16.637	36,490,927.84	90
Ethyl palmitoleate	C18 H34 O2	282.2548	20.987	35,975,153.82	93.2
NP-001596	C16 H30 O4	308.1952	18.984	35,777,548.18	94.9
NP-011548	C18 H34 O3	280.239	21.041	34,105,859.15	75.6
4-Phenylbutyric acid	C10 H12 O2	164.0831	11.577	33,493,328.71	76.5
Oleamide	C18 H35 N O	281.2706	20.551	32,293,321.14	91.8
Tetranor-12R-HETE	C16 H26 O3	248.1766	14.077	32,107,702.51	71.9
Palmitoleic acid	C16 H30 O2	254.2235	20.687	31,698,429.00	70.8
4-hydroxy-6-[2-(2-methyl-1,2,4a,5,6,7,8,8a-octahydronaphthalen-1-yl)ethyl]oxan-2-one	C18 H28 O3	314.1846	16.588	31,279,593.58	86.6
1-Linoleoyl glycerol	C21 H38 O4	354.2756	17.366	31,243,191.82	92.3
n-Pentyl isopentyl phthalate	C18 H26 O4	323.2083	18.021	31,197,110.75	67.8
NP-001596	C16 H30 O4	268.2028	16.258	30,927,401.68	73.8
Dibenzylamine	C14 H15 N	197.1198	7.466	30,636,932.36	91.6
Ouabain	C29 H44 O12	622.2406	19.208	30,368,965.79	79.6
NP-014287	C18 H32 O3	318.216	18.515	30,124,986.19	93.2
(1aR,1bR,2R,3R,7R,7aS)-1b,2-dimethyl-7a-(prop-1-en-2-yl)-1aH,1bH,2H,3H,4H,5H,7H,7aH-naphtho[1,2-b]oxirene-3,7-diol	C15 H22 O3	232.1455	12.819	29,968,764.20	74.6
Choline	C5 H13 N O	103.0996	9.85	28,794,445.64	97.3
OPEO	C16 H26 O2	250.1923	18.372	28,609,170.94	61.8
4-Methylbenzophenone	C14 H12 O	196.0881	15.64	28,287,214.04	92.5
(±)8-HEPE	C20 H30 O3	300.2076	16.047	27,816,026.26	92.2
(±)11(12)-EET	C20 H32 O3	302.2233	17.364	27,544,881.86	93.1
(4aR,5R,6R)-6-hydroxy-4a,5-dimethyl-3-(prop-1-en-2-yl)-2,4a,5,6,7,8-hexahydronaphthalen-2-one	C15 H20 O2	214.135	14.449	27,409,945.13	63.8
2,3-dihydroxypropyl 12-methyltridecanoate	C17 H34 O4	302.2444	16.302	27,286,206.71	71.5
Palmitoleic acid	C16 H30 O2	254.2235	16.593	25,831,647.73	89.4
12-Oxo phytodienoic acid	C18 H28 O3	292.2026	16.524	25,662,668.92	62.7
Cafestol	C20 H28 O3	316.2003	15.864	25,328,627.10	89.3
[6-Hydroxy-1-(hydroxymethyl)-1,4a-dimethyl-5-(2-oxo-2-pyrrolidin-1-ylethyl)-2,3,4,5,6,7,8,8a-octahydronaphthalen-2-yl] N-[3-(trifluoromethyl)phenyl]carbamate	C27 H37 F3 N2 O5	508.263	14.695	24,803,082.67	66.8
Eicosapentaenoic acid	C20 H30 O2	308.2339	20.606	24,591,569.01	78.1
R-Palmitoyl-(2-methyl) ethanolamide	C19 H39 N O2	313.2967	21.592	23,630,078.82	91.6

2-(2,4-dichlorophenyl)-4,4,7,9-tetramethyl-4,5-dihydro-3H-naphtho[1,2-d]imidazole	C21 H20 Cl2 N2	370.094	12.163	23,533,439.36	72.4
Ageratriol	C15 H24 O3	234.1611	12.76	23,504,570.02	80.9
(3aR,4aS,5R,8S,9aR)-5-hydroxy-4a,8-dimethyl-3-methylidene-2H,3H,3aH,4H,4aH,5H,6H,8H,9H,9aH-azuleno[6,5-b]furan-2,6-dione	C15 H18 O4	244.1091	14.233	23,426,676.24	71.9
(1aR,1bR,2R,3R,7R,7aS)-1b,2-dimethyl-7a-(prop-1-en-2-yl)-1aH,1bH,2H,3H,4H,5H,7H,7aH-naphtho[1,2-b]oxirene-3,7-diol	C15 H22 O3	232.1455	13.406	23,234,665.52	82.3
Ouabain	C29 H44 O12	622.2406	20.998	23,194,206.37	83.3
ethyl 2-(methylthio)-4-tetrahydro-1H-pyrrol-1-yl-8-(trifluoromethyl)quinoline-3-carboxylate	C18 H19 F3 N2 O2 S	384.1096	12.099	22,988,983.05	73.6
NP-004713	C15 H24 O2	218.1662	18.446	22,839,809.05	93.6
(1aR,1bR,2R,3R,7R,7aS)-1b,2-dimethyl-7a-(prop-1-en-2-yl)-1aH,1bH,2H,3H,4H,5H,7H,7aH-naphtho[1,2-b]oxirene-3,7-diol	C15 H22 O3	232.1454	11.357	22,776,743.89	85.4
Eicosapentaenoic acid ethyl ester	C22 H34 O2	330.2546	20.376	22,099,138.42	88.9
NP-020214	C16 H32 O4	270.2183	17.161	21,808,214.15	79
Eicosapentaenoic acid	C20 H30 O2	302.2233	19.79	21,421,271.65	92.7
Levalbuterol	C13 H21 N O3	261.1354	13.431	20,606,367.65	92.4
Linolenic acid ethyl ester	C20 H34 O2	306.2545	20.074	20,450,089.14	94.8
4-{[(4,6-Dimethoxypyrimidin-2-yl)amino]methylidene}-2-phenyl-4,5-dihydro-1,3-oxazol-5-one	C16 H14 N4 O4	326.1044	18.031	20,248,246.76	68.5
Ethyl oleate	C20 H38 O2	310.2857	21.707	19,935,241.14	86
Benzophenone	C13 H10 O	182.0725	14.41	19,870,753.40	96.9
octadec-9-ynoic acid	C18 H32 O2	262.2285	17.367	18,964,856.34	96.9
(3aS,5aS,9bR)-5a,9-dimethyl-3-methylidene-2H,3H,3aH,4H,5H,5aH,6H,7H,8H,9bH-naphtho[1,2-b]furan-2,5-dione	C15 H18 O3	228.1141	15.997	18,954,242.24	81.2
Docosahexaenoic acid	C22 H32 O2	328.2388	19.433	18,184,819.14	74.3
Cetrimonium	C19 H41 N	283.3227	26.392	18,033,386.90	90.6
3-(3,4-dimethoxyphenethyl)-2-[(4-isopropylphenyl)imino]-1,3-thiazolan-4-one	C22 H26 N2 O3 S	398.169	17.063	17,943,409.82	65.4
(+/-)-C75	C14 H22 O4	236.1402	9.957	17,802,168.49	84.1

(1aR,1bR,2R,3R,7R,7aS)-1b,2-dimethyl-7a-(prop-1-en-2-yl)-1aH,1bH,2H,3H,4H,5H,7H,7aH-naphtho[1,2-b]oxirene-3,7-diol	C15 H22 O3	232.1454	11.539	17,670,064.36	85.4
3,5-di-tert-Butyl-4-hydroxybenzaldehyde	C15 H22 O2	234.1611	16.951	17,509,512.15	95
Palmitoyl ethanolamide	C18 H37 N O2	299.2812	20.317	17,492,831.93	97.8
Tetranor-12R-HETE	C16 H26 O3	248.1766	14.265	17,087,612.46	71.9
4-Phenylbutyric acid	C10 H12 O2	164.0831	21.361	16,843,242.68	96.1
5-(furan-3-yl)-1',9'-dihydroxy-12'-(hydroxymethyl)-6'-methyl-3'-oxaspiro[oxolane-3,7'-tricyclo[6.3.1.0â�ÂaÂ?]dodecane]-2-one	C20 H26 O7	400.1408	16.405	16,574,129.08	60.5
Oxethazaine	C28 H41 N3 O3	467.3231	15.118	16,405,749.44	62
NP-016582	C20 H35 N O	305.2705	19.858	16,337,402.48	78.1
n-Pentyl isopentyl phthalate	C18 H26 O4	323.2083	17.911	16,298,012.70	67.8
Sphingosine (d18:1)	C18 H37 N O2	321.2654	20.015	16,138,319.09	78.7
4-(3-methoxy-5,6-dihydrobenzo[c]acridin-7-yl)morpholine	C22 H22 N2 O2	692.3268	16.742	15,941,236.73	71.2
(4aR,5R,6R)-6-hydroxy-4a,5-dimethyl-3-(prop-1-en-2-yl)-2,4a,5,6,7,8-hexahydronaphthalen-2-one	C15 H20 O2	214.135	12.673	15,882,573.51	63.7
(-)-Strychnine	C21 H22 N2 O2	334.1743	13.152	15,540,877.24	80.7
NP-004713	C15 H24 O2	218.1662	18.809	15,521,801.11	95.1
Arachidonic acid	C20 H32 O2	304.2391	20.726	15,378,473.45	95.6
3,4-Diphenylpyrimido[4',5':4,5]thieno[2,3-c]pyridazin-8(7H)-one	C20 H12 N4 O S	356.0783	16.279	15,308,423.13	67
2-Arachidonoyl glycerol	C23 H38 O4	378.2755	17.305	14,960,482.55	89
4-Phenylbutyric acid	C10 H12 O2	164.0831	13.211	14,944,621.17	76.8
Testosterone decanoate	C29 H46 O3	442.3432	19.83	14,601,900.81	73
(5E)-7-methylidene-10-oxo-4-(propan-2-yl)undec-5-enoic acid	C15 H24 O3	274.1558	14.886	14,357,946.57	71
γ-Linolenic acid ethyl ester	C20 H34 O2	312.2651	17.72	13,897,675.48	64.4
Choline	C5 H13 N O	103.0996	10.194	13,726,162.09	97.2
6-Hydroxy-1-(hydroxymethyl)-5-{2-[2-(hydroxymethyl)-1-pyrrolidinyl]-2-oxoethyl}-1,4a-dimethyldecahydro-2-naphthalenyl phenylcarbamate	C27 H40 N2 O6	510.2786	15.669	12,500,838.24	81.4
Nor-9-carboxy-δ9-THC	C21 H28 O4	344.195	19.475	12,426,002.76	83
6-Aminocaproic acid	C6 H13 N O2	131.0942	26.449	12,331,286.20	76.4

NP-004713	C15 H24 O2	218.1662	16.537	11,878,843.21	89.4
NP-001596	C16 H30 O4	268.2028	16.984	11,838,885.59	80.4
(2E)-5-[(8aS)-2,5,5,8a-tetramethyl-3-oxo-3,4,4a,5,6,7,8,8a-octahydronaphthalen-1-yl]-3-methylpent-2-enoic acid	C20 H30 O3	340.2023	21.194	11,545,932.49	72.9
Choline	C5 H13 N O	103.0996	10.967	11,359,646.88	96.7
Tetranor-12R-HETE	C16 H26 O3	248.1766	13.864	10,238,258.33	72.3
Palmitoleic acid	C16 H30 O2	254.2235	17.969	10,100,783.45	87.7
NP-011548	C18 H34 O3	280.239	17.659	10,070,685.41	82.8
Propionylpromazine	C20 H24 N2 O S	340.1637	17.805	9,806,912.19	67.1
(±)8-HEPE	C20 H30 O3	300.2076	16.796	9,756,031.63	88
Di(2-ethylhexyl) phthalate	C24 H38 O4	390.2753	23.138	9,589,300.00	99.2
γ-Linolenic acid ethyl ester	C20 H34 O2	312.2651	16.315	9,512,652.76	67.9
Sedanolide	C12 H18 O2	176.1194	20.548	9,339,555.94	70.2
Arachidonoyl amide	C20 H33 N O	303.2549	19.703	9,113,695.23	84.6
Tetranor-12(S)-HETE	C16 H26 O3	248.1766	16.962	8,865,034.10	72.2
8Z,11Z,14Z-Eicosatrienoic acid	C20 H34 O2	306.2545	18.548	8,618,378.15	94.9
Oleyl anilide	C24 H39 N O	363.3121	21.448	8,239,070.91	66.6
Palmitoleic acid	C16 H30 O2	254.2235	16.814	5,812,211.91	73.4

Total Ion Chromatogram

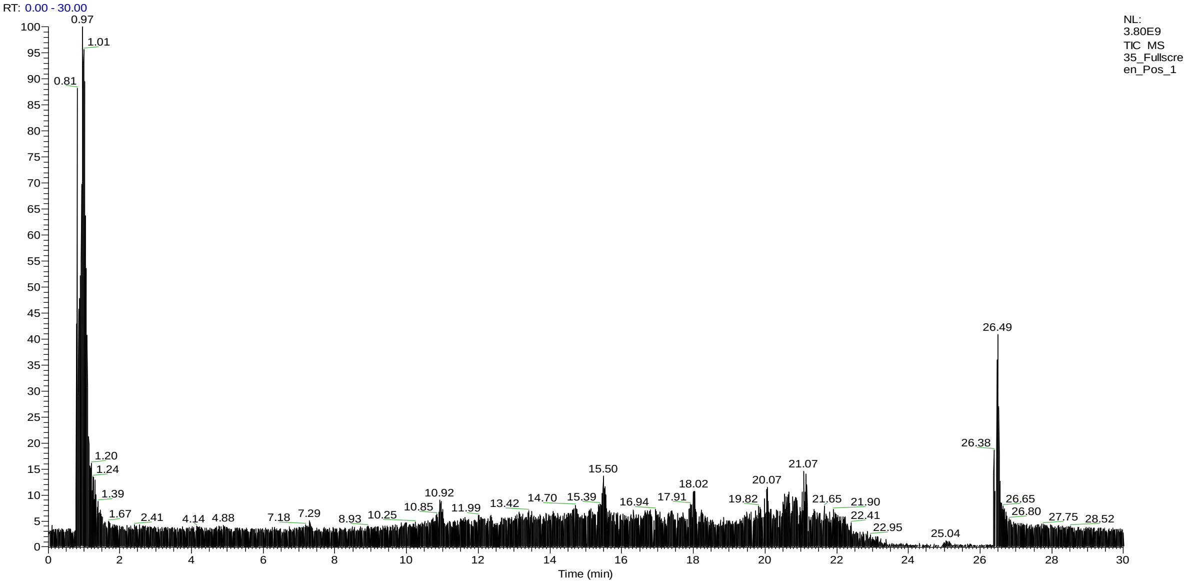

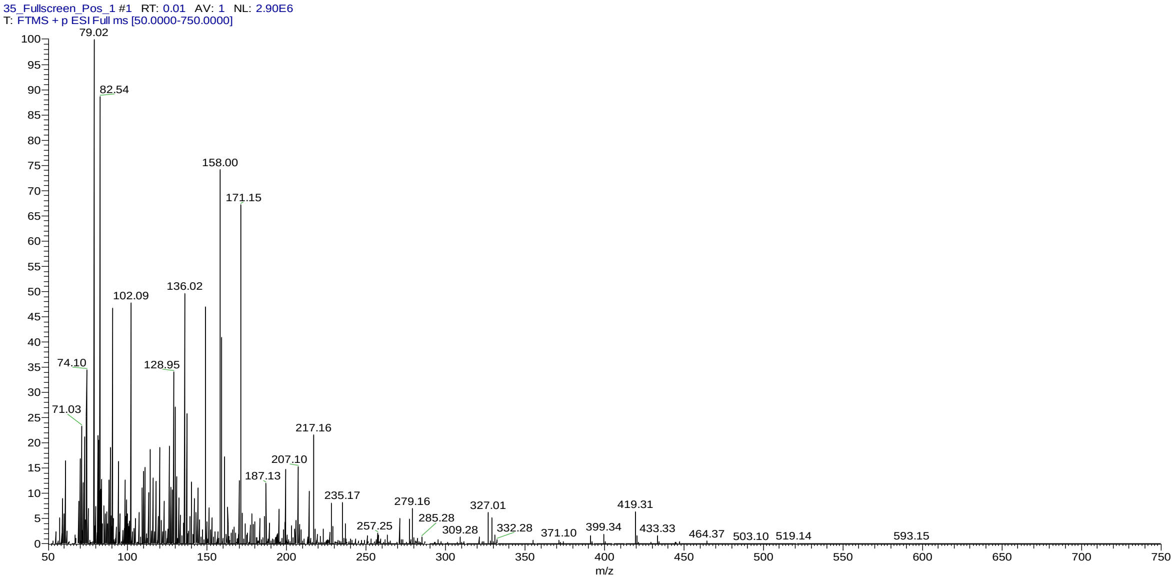

Supplement: Supplementary 2 — Metabolomic profiling of sea grape extract. [file Data_Sheet_2.PDF]
